# Assessment of Hip and Lumbar Spine Range of Motion After Total Hip Arthroplasty Using a Single Camera Markerless System

**DOI:** 10.7759/cureus.65875

**Published:** 2024-07-31

**Authors:** Anastasios G Roustemis, Panagiotis Gavriil, Apostolos Z Skouras, Dimitra Melissaridou, Spyridon Sioutis, Ioannis Trikoupis, Vasileios Karampikas, Konstantinos Avgerinos, Pavlos Altsitzioglou, Panagiotis Koulouvaris, Panayiotis J Papagelopoulos, Olga Savvidou

**Affiliations:** 1 1st Department of Orthopaedic Surgery, National and Kapodistrian University of Athens School of Medicine, Athens, GRC; 2 1st Department of Orthopaedic Surgery/Sports Excellence, National and Kapodistrian University of Athens School of Medicine, Athens, GRC

**Keywords:** markerless motion capture, rehabilitation, monocular human motion capture, joint kinematics, total hip replacement

## Abstract

Background: Total hip arthroplasty (THA) is one of the most cost-effective and successful procedures in orthopedics. However, assessing the post-operative range of motion (ROM) remains a challenge due to the limitations of traditional measurement methods. This study aimed to evaluate hip and spine ROM post-operatively and single-leg balance, using a single-camera markerless motion capture system, and compare outcomes with pre-operative ROM and with an age-matched healthy control group.

Methods: An interventional study was conducted from January 2018 to December 2021. Twenty patients with hip osteoarthritis underwent THA and were assessed using a single-camera markerless system (Kinetisense software). Measurements were taken one month pre-operatively and one year post-operatively.

Results: Significant improvements were observed in hip and lumbar spine ROM variables after THA. The most notable enhancements were in hip and spinal flexion. Compared to the control group, the THA group showed minor deficits in hip ROM, particularly in external rotation. Single-leg balance demonstrated improved stability post-operatively.

Conclusions: The single-camera markerless motion capture system offers a promising alternative for assessing hip and lumbar spine ROM, presenting potential advantages over manual goniometry and traditional 3D motion capture systems. Using this system for the evaluation of patients after THA, it seems that THA significantly enhances hip and lumbar spine ROM. Future research should focus on validating the accuracy of markerless systems.

## Introduction

Total hip arthroplasty (THA) has been characterized as the operation of the century [1,2]. THA is a successful orthopedic procedure that improves the quality of life of patients with end-stage arthritis. The most significant factors contributing to the enhancement of patients' quality of life after THA are the alleviation of pain and the improvement in hip ROM [3]. However, studies identified that at least 7% of patients remain unsatisfied after surgery [4].

Aligning patient expectations with realistic surgical outcomes is crucial for maximizing post-operative satisfaction. Ghomrawi et al. highlighted that over 50% of patients have greater expectations than their surgeon for high-level activities and extensive ROM restoration post-operatively [5].

Multiple surgery and rehabilitation-related factors can contribute to decreased post-operative ROM [6,7]. Several post-operative outcomes, particularly in terms of functional recovery, are predominantly influenced by the hip ROM. Moreover, ROM remains a commonly assessed outcome measure in exercise intervention studies [8].

Traditionally, the assessment of hip ROM has been reliant on manual goniometric measurements, an approach that is often subject to inter-rater variability and potential inaccuracies [9-11]. These evaluations, predominantly passive, are usually conducted with the patient in bed and preset standard positions. However, the clinical realm today acknowledges the superiority of dynamic movement assessment in capturing true functional potential [12].

The gold standard for functional assessment is 3D marker-based motion analysis systems that assess spatiotemporal characteristics, kinematics, and kinetics during gait and other tasks, offering insights into acceptable functional ability [13,14]. However, these systems have several disadvantages such as high cost, time-consuming procedures, and the necessity for skilled personnel [12-14]. The demand for practical functional movement assessment tools led to development of many technologies, such as the markerless motion capture system. The advent of markerless motion capture systems offers a practical and user-friendly alternative for clinicians.

This study aims to evaluate hip and spine ROM in standard weight-bearing movements, and single-leg balance, by comparing the operated limb with the THA with the non-operated limb and with the ROM of the hip in an age-matched healthy control group.

## Materials and methods

An interventional study was conducted between January 2018 to December 2021. Twenty patients with hip osteoarthritis (OA) planning to undergo THA were recruited. Patients underwent THA at an orthopedic department in a tertiary hospital. A sample of 20 voluntary patients was sequentially selected from our outpatient clinics and placed on the waiting list for hip OA surgery, all meeting the same inclusion and exclusion criteria. All participants provided written consent prior to inclusion. The study protocol received approval by the hospital’s ethics and scientific committee (protocol number 0112/2021). Measurements and data collection were carried out by a team of five resident orthopedic surgeons and a physical therapist under the supervision of two senior orthopedic surgeons.

The surgeries were performed by two hip surgeons, using a posterolateral approach with a cementless cup and stem (Medacta hip replacement system, Switzerland). Preoperative screening included clinical examination, plain radiographs, and computed tomography of the hip. Radiographic classification of OA was assessed using the Kellgren and Lawrence system.

Patients were allowed weight-bearing immediately post-surgery. The hospital's physiotherapy department undertook normal gait retraining, according to standard hospital practice, and patients then continued their rehabilitation at a primary care setting of their preference.

All enrolled patients had to fulfill eligibility criteria. Participants need to be aged between 65 and 85, diagnosed with primary hip OA, and able to adhere to study requirements including multiple imaging and motion analyses were ambulant pre-operative.

Exclusion criteria were patients with a history of major lower extremity orthopedic surgeries, lower limb joint pain or dysfunction irrelevant to hip OA, current low back pain, severe spinal deformities, rheumatic conditions, advanced neurological disorders, class III obesity (body mass index - BMI > 40 m^2^/kg), any condition impairing walking ability, except for primary OA, or difficulty to understand and read the study protocol instructions and consent form.

Single camera markerless motion capture system

Hip and spine ROM and single-leg balance were assessed using the Intel® RealSense™ D435 camera (Mission College Blvd., Santa Clara, CA, US) and analyzed with the Kinetisense software (Medicine Hat, AB T1B 0M9, Canada). Assessments were performed in a room free of objects with enough space to conduct movements with restriction. The camera was placed 1 to 1.5 m off the ground and 2 to 2.5 far from the participants. They were also instructed to wear colored or black clothes to contrast with the wall. To avoid artifacts, no external support was permitted. All measurements were conducted one month pre-operatively and one year post-operatively.

The ROM was measured for lumbar spine flexion, extension, and lateral flexion. Lumbar spine flexion was measured through forward bending.

Additionally, hip motion (flexion-extension, abduction-adduction, internal-external rotation) was assessed. Hip assessment was performed bilaterally. All measurements were taken from a standing position, perpendicular to the camera. Then, participants balanced on each leg for 20 seconds, maintaining their gaze with their eyes open. Each leg was tested twice, with the best performance selected for analysis. The sway patterns during the balance test were further analyzed using the Kinetisense software.

Statistical analysis

For descriptive statistics, results are expressed as means ± standard deviation. Spine (flexion, extension, and lateral flexion), hip ROM (flexion-extension, abduction-adduction, hip internal-external rotation), and single-leg balance have been defined as dependent variables. The assumption of normality was tested with the Shapiro-Wilk test. In cases where the data did not follow a normal distribution and where outliers were detected in the groups under examination, the non-parametric Wilcoxon signed-rank test was used to compare the operated limb with the non-operated limb. A box plot was used to detect outliers in the examined sample. Any participant who returned a value more than 1.5 times the interquartile range is characterized as an outlier. Paired t-test or Wilcoxon test for dependent samples were used to compare pre-operative and post-operative data, respectively for parametric or non-parametric data. The t-test or the Mann-Whitney U test for independent samples was used to compare patients with healthy controls, respectively for parametric and non-parametric data. The Welch t-test was used to determine whether there were differences in the assumption of homogeneity of variances as tested by Levene's test. The significance level was set as α ≤ 0.05. IBM SPSS Statistics for Windows, Version 18 (Released 2009; IBM Corp., Armonk, New York, United States) was used for the statistical analyses.

## Results

Eleven male and nine female patients with hip OA enrolled in the study. The mean age of the patients was 70.3 ± 4.58 years, and the BMI was 29.35 ± 2.13 kg/m^2^. Six patients presented with a grade 4 and four patients with a grade 3 of Kellgren and Lawrence scale. Half sample underwent THA on the right side, and half on the left. Twenty voluntary patients, sequentially selected from our outpatient clinics and meeting the same inclusion and exclusion criteria, were placed on the waiting list for hip osteoarthritis surgery and used as a reference group.

One year after THA, statistically significant improvement was demonstrated in all lumbar spine and hip ROM variables (Table 1).

**Table 1:** Hip range of motion (ROM) pre-operative and post-operative for operated and non-operated limbs. The data is presented as mean ± standard deviation. ROM, range of motion; THA, total hip arthroplasty. Statistical tests used: Paired t-test or Wilcoxon test for dependent samples, t-test or Mann-Whitney U test for independent samples, Welch t-test for homogeneity of variances. p ≤ 0.05 considered significant.

ROM measurements (deg)	Preop (deg ± SD)	Postop (deg ± SD)	p-value
Lumbar spine ROM			
Flexion	61.88 ± 5.92	78.57 ± 6.19	< 0.001
Extension	22.69 ± 1.70	25.29 ± 0.97	0.002
Lateral flexion (L)	13.54 ± 1.81	15.09 ± 1.62	0.006
Lateral flexion (R)	14.07 ± 1.29	16.35 ± 0.92	< 0.001
Hip ROM			
Flexion non-operated	82.22 ± 5.50	84.33 ± 5.08	< 0.001
Flexion operated	65.84 ± 9.89	81.87 ± 7.87	0.01
Extension non-operated	28.67 ± 3.97	30.60 ± 4.76	0.005
Extension operated	26.34 ± 3.88	28.51 ± 5.93	0.008
Abduction non-operated	35.29 ± 2.00	37.44 ± 3.18	0.008
Abduction operated	30.60 ± 3.95	36.68 ± 7.11	0.003
Adduction non-operated	19.00 ± 1.89	20.72 ± 1.60	< 0.001
Adduction operated	21.13 ± 2.73	22.45 ± 2.80	< 0.001
External rotation non-operated	22.14 ± 4.20	26.97 ± 3.04	< 0.001
External rotation operated	21.50 ± 3.55	27.03 ± 2.34	< 0.001
Internal rotation non-operated	29.66 ± 3.22	35.20 ± 3.44	0.003
Internal rotation operated	28.39 ± 5.47	36.73 ± 3.67	< 0.001

The greatest change occurred in the sagittal plane, and in particular in spinal flexion, by 16.69° ± 6.98° (p < 0.001), while lateral flexion, regardless of side, showed the least improvement. Accordingly, at the hip joint, flexion revealed the greatest postoperative improvement, with an increase of 16.03° ± 10.92° (p < 0.001) for the operated hip and 14.55° ± 7.23° (p < 0.001) for the non-operated. The affected side had a significantly greater extension postoperatively, approaching the healthy population. The analysis shows an increase in hip ROM rotations, external and internal rotation, of more than 10° for both limbs.

Compared with the control group, the THA group showed small deficits in hip ROM one year after surgery (Table 2).

**Table 2:** Comparison of Hip ROM between THA patients and age-matched healthy controls. The data is presented as mean ± standard deviation. ROM, range of motion; THA, total hip arthroplasty. Statistical tests used: Paired t-test or Wilcoxon test for dependent samples, t-test or Mann-Whitney U test for independent samples, Welch t-test for homogeneity of variances. p ≤ 0.05 considered significant.

ROM measurements (deg)	THA (deg ± SD)	Healthy (deg ± SD)	p-value
Lumbar spine ROM			
Flexion	78.57 ± 6.19	81.70 ± 7.81	0.334
Extension	25.29 ± 0.97	26.19 ± 1.02	0.059
Lateral flexion (L)	15.09 ± 1.62	15.65 ± 1.33	0.407
Lateral flexion (R)	16.35 ± 0.92	17.11 ± 0.98	0.09
Hip ROM			
Flexion non-operated / right	84.33 ± 5.08	86.43 ­± 3.79	0.064
Flexion operated / left	81.87 ± 7.87	83.83 ± 8.23	0.593
Extension non-operated / right	30.60 ± 4.76	32.12 ± 4.64	0.478
Extension operated / left	28.51 ± 5.93	30.30 ± 5.15	0.481
Abduction non-operated / right	37.44 ± 3.18	38.23 ± 2.51	0.544
Abduction operated / left	36.68 ± 7.11	37.43 ­± 6.55	0.81
Adduction non-operated / right	20.72 ± 1.60	21.34 ± 1.12	0.33
Adduction operated / left	22.45 ± 2.80	22.05 ± 1.53	0.073
External rotation non-operated / right	26.97 ± 3.04	34.81 ± 2.98	< 0.001
External rotation operated / left	27.03 ± 2.34	35.28 ± 3.20	< 0.001
Internal rotation non-operated / right	35.20 ± 3.44	38.92 ± 1.48	0.006
Internal rotation operated / left	36.73 ± 3.67	39.21 ± 2.33	0.091

For the frontal plane, maximal abduction and adduction of the affected side were comparable to the control group for both limbs (p > 0.05). The same was observed for lateral bending of the trunk, regardless of side. In the sagittal plane, neither hip extension nor hip flexion showed a statistically significant difference between THA and control groups. Conversely, hip external rotation for the control group was statistically significantly higher than the THA group for both limbs (p < 0.001).

The postural sway during single-leg balance showed a statistically significant decrease in both lower limbs (operated and non-operated), demonstrating improved stability one year after surgery. The operated lower extremity had a greater change of 6.74 ± 4.17 mm^2^ (p = 0.001), compared to the non-operated limb (2.3 ± 1.32 mm^2^, p < 0.001) (Table 3).

**Table 3:** Single-leg balance assessment preoperative and postoperative for operated and non-operated limbs. The data is presented as mean ± standard deviation. ROM, range of motion; THA, total hip arthroplasty. Statistical tests used: Paired t-test or Wilcoxon test for dependent samples, t-test or Mann-Whitney U test for independent samples, Welch t-test for homogeneity of variances. p ≤ 0.05 considered significant.

Postural sway (mm^2^)	Preop	Postop	p-value
Non-operated limb	39.65 ± 9.23	37.34 ± 9.34	0.001
Operated limb	36.93 ± 7.87	30.19 ± 8.64	< 0.001

## Discussion

The aim of the study was to investigate hip and spine ROM one year after THA compared to pre-operative ROM and to that of healthy controls using a single camera of motion capture. Τhe post-operative ROM was comparable to that of healthy controls for hip and spine movements. Our findings indicate that THA significantly improves hip and spine ROM. While markerless motion capture systems employing only one camera have yet to be thoroughly validated for accuracy, they show promise in addressing the limitations of manual goniometric measurements and traditional 3D motion capture systems.

Goniometry (manual and digital) assessing the range of passive motion has several disadvantages and pitfalls. Leighton flexometers, inclinometers, and tape measures are used for some specific body parts, such as lumbar spine flexion through the Schober test. There is a high variability in the position of measurement. For example, hip internal/external rotation can be measured from a sitting, supine with tibia outside of the bed, and prone position. Although Kouyoumdjian et al. found no statistically significant differences between these three positions among 120 healthy adults [15], the total hip rotation ROM was on average 68.1 degrees for supine, 77.1 degrees for prone, and 78.5 degrees for seated with the hip in flexion. Another drawback of passive ROM measurement is in intra- and inter-rater reliability and validity [9-11]. This difficulty may also arise from challenges in stabilizing and controlling pelvic tilt and rotation during passive measurements [9]. Moreover, the range of passive motion is significantly larger than active motion in the hip joint for older adults over 70 years old [16], making it difficult to transfer passive ROM values into clinical practice.

In our study, we observed that hip flexion in older adults was lower than textbook reference values [17], with a notable discrepancy when compared with other research, such as the work of James and Parker [16], who reported approximately 100 degrees of flexion in contrast to our finding of 85 degrees. The variation may be attributable to different testing positions; assessment was in standing, not supine as in James and Parker's study [16]. Additionally, total hip rotation in our sample was less than referenced in other position-comparative studies [15], and patients after THA showed a pronounced reduction in hip rotation, especially externally, highlighting the importance of targeted rehabilitation. The assessment of active hip ROM is crucial after THA due to its role in functional recovery, yet we find that even healthy older adults show decreased ROM [18], which may not fulfill the demands of daily activities, like tying shoes which require up to 120 degrees of hip flexion [19]. Moreover, there is a documented decline in all hip motions over time, with women generally exhibiting greater mobility [16]. Balance is also critical after THA; our research found improvements in single-leg balance, yet not to the level of healthy adults, emphasizing the need for specialized balance training, as well [20].

Traditional 3D marker-based motion capture systems, while accurate, are costly, require extensive setup, space, and specialized staff training, and can be time-consuming, presenting challenges for facilities with high patient turnover and limited resources [21]. Consequently, markerless motion capture technologies have begun to emerge as a solution to these constraints, offering a more streamlined and accessible approach to assessing active ROM in clinical settings [22].

Markerless motion capture is a technique that utilizes conventional video footage and often depends on software using deep learning techniques, specifically pose estimation algorithms, to analyze and interpret the body positions of individuals in each frame of the video or across many camera recordings [22]. This approach offers advantages in terms of cost-effectiveness and spatial efficiency [22]. State-of-the-art markerless systems with multiple cameras and complex software are comparable in accuracy to 3D marker-based motion capture systems [23]. However, while these markerless systems are practical compared to marker-based, they are still at similar cost.

The use of 2D single-camera markerless motion capture, which consists of only one camera, involves the extraction of joint center positions from a picture or video via the application of 2D pose estimation algorithms [22]. Current research suggests an acceptable accuracy and reliability of single-camera markerless motion capture for joints with large ROM, such as knee and shoulder joints [24-26]. Most studies use the Microsoft Kinect video capture camera [27], as we did in our research. Given this, the system might be a practical tool to monitor disease progression or quantify improvements in spine and hip joint motion after THA. Thus, its use could lead to impactful applied research in clinical practice, offering objective measurements with low-cost instrumentation.

Limitations

This study has several limitations. Firstly, the sample size was relatively small, which may affect the generalizability of the findings. Secondly, the use of a single-camera markerless motion capture system, although practical and user-friendly, may not be as accurate as traditional multi-camera systems. Additionally, the study did not include long-term follow-up beyond one year post-operatively, which limits the understanding of the durability of the improvements observed. Lastly, the control group consisted of healthy older adults, which may not perfectly match the patient population undergoing THA in terms of physical activity levels and other health conditions. Future research should aim to include larger sample sizes, longer follow-up periods, and possibly multiple camera systems to enhance the validity and reliability of the findings.

## Conclusions

A markerless motion capture system is a useful tool for assessing ROM after THA. One year post-operatively, there was a marked improvement in both hip and lumbar spine ROM in all patients, with the most significant enhancement observed in flexion of the hip and spine. While the THA group exhibited minor deficits in hip ROM when compared to the age-matched control group, especially in hip external rotation, the differences in most movements were not statistically significant. This indicates that THA effectively restores ROM to levels similar to those of a healthy population. The single-camera markerless motion capture system offers a user-friendly alternative for ROM assessments that is not user-dependent compared to manual goniometry and is less expensive than marker-based 3D motion capture systems. Future research should aim to validate the accuracy of markerless motion capture systems further.
